# The Internet as a source of reproductive health information among adolescent girls in an urban city in Nigeria

**DOI:** 10.1186/1471-2458-7-354

**Published:** 2007-12-20

**Authors:** Williams E Nwagwu

**Affiliations:** 1 Africa Regional Centre for Information Science, University of Ibadan, Ibadan, Oyo State, Nigeria

## Abstract

**Background:**

There exists some research evidence regarding how adolescents utilize the Internet for health information seeking purposes. The purpose of this study is to understand how in-school and out-of-school adolescent girls in Owerri, Nigeria use online resources to meet their reproductive health information needs. The result could be considered very crucial in assessing the potential role of the Internet in providing health information to adolescent girls in a typical Nigerian urban city.

**Methods:**

A questionnaire was used to collect data from 1011 adolescent girls in selected secondary schools in the communities, and also from 134 out-of-school girls selected from the same communities.

**Results:**

More than 73% of the girls reported having ever used the Internet; more than 74% and 68% of them being in-school and out-of-school respectively. The in-school girls (43.9%) reported having home access more than the out-of-school (5.6%) although the out-of-school have used the Internet for finding reproductive and related information more than the in-school. While parents (66.22%) and teachers (56.15%) are the two sources most used to the in-school girls, friends (63.18%) and the Internet (55.19%) were reported by the out-of-school youth as the two most used sources of information to them.

**Conclusion:**

The Internet is not a first choice of source of reproductive health information for both the in-school and out-of-school adolescent girls in Owerri, Nigeria. The source is however, more commonly used by the out-of-school than the in-school, but the in-school have a more favorable assessment of the quality of information they obtain from the Internet.

## Background

Adolescence is a critical period of human development often characterized by confusion, mixed interpretation and understanding of adult behaviour and environment, exuberance and a penchant for experimentation, especially with drugs, alcohol and sex [1]. Of all challenges, those associated with sexual maturation are the most distinctive as well as the most problematic [2]. This stage of development is accompanied by an upsurge of sex drives, the development of sexual values, and the initiation of sexual behavior [3]. The key concern about the health of young people is the extent to which they have access to resources that promote their development. Access to information and communication services is now seen as a universal right, and the United Nations is advocating for a global initiative for such access within this decade [4]. The resources that adolescents need include: access to education, information and services; resources that reside in a stable and supportive structure such as the family; resources contained within policy-making and decision-making processes, and many young people do not have access to these facilities. To improve young people's access to these resources, new strategies that are attractive to the youth are beginning to emerge, and they make use of the power, creativity and enthusiasm of adolescents. This is where information technology, such as the Internet, is expected to play a critical role as a source of information.

A cursory scan of Owerri shows that, as probably in many other Nigerian cities, Internet surfing by both boys and girls for various purposes is a very common practice. It is not uncommon to spot school girls and boys in their uniforms in the cybercafés even during school hours. Given the harsh social and economic conditions in Nigeria, many youth are out-of-school for either deviant reasons, inability of parents to maintain them, or they have concluded their secondary schooling but are yet to be engaged in any meaningful activities. There are initiatives that have recognised and are advocating for the use of the Internet as a reproductive health information source for youth in Nigeria. But empirical studies on how the youth in Owerri rely on online resources for reproductive health information have not been established, and this is the focus of this study.

Many people are optimistic about the Internet as a source of information to young people. Some assert that a primary role of the Internet is to deliver information and improve the health of populations, especially in developing countries [5,6]. Moreover, several studies have shown that young people are more likely to go online than their older counterparts. Although concern is raised about the quality of online health information [7], the nature of this technology, including ease of access, anonymity and non-punitive attributes, make it an attractive information source to the youth, especially for sensitive health issues [8-10]. This observation can be accommodated by the Uses and Gratification Theory, which emphasises the significance of people's value for independence in the search and use of information and communication media.

### Uses and gratification theory: A theoretical background

Uses and Gratifications (U&G) theory due to Blumler & Katz (1974) [11] is fast becoming an influential tradition in media research because it focuses on why people use the media rather than on the content of the media. The theory assumes that the individual user of the media is in control, active, and goal-directed, as opposed to simply receiving media messages. The media user has the power to choose what is considered needful. U&G can be seen as part of a broader trend among media researchers, which is more concerned with what people themselves do with media, allowing for a variety of responses and interpretations. The media user consciously or subconsciously takes the initiative to link gratification needs with his or her media choice and use, from among alternative media and other available sources based on the fact that such is able to decide on the information required, select such information and use it [11,12]. U & G views the media in terms of the gratification of social or psychological needs of the individual [11]. An empirical study in the U & G tradition typically involves audience members completing a questionnaire about why they use the media.

## Literature review

### Reproductive health

The UN Conference on Population and Development recently defined health as:

*... a state of complete physical, mental and social well being and not merely the absence of disease or infirmity in all matters relating to the reproductive system and to its functions and processes. .... [It] implies that people are able to have a satisfying and safe sex life and that they have the capability to reproduce and the freedom to decide if, when and how often to do so *[13].

Sexuality refers to the social expression of one's social and biological being through mannerisms, mode of dressing, interaction patterns, and physical intercourse. Implicit in these definitions are the rights of persons to be informed and to have access to safe, effective, affordable, and acceptable as well as the right to appropriate health-care services. Sexual expression is an essential component of healthy human development for individuals of all ages. In general, an adolescent's age, socio-economic status, family atmosphere, sexual orientation, religious commitment, and individual life experiences are all factors that can exert an influence on whether, when, and how she or he will be sexually active. Sexual expression can be either positive or negative depending on the context.

### Adolescence, reproductive health information and the Internet

Adolescence is that period, between puberty and adulthood. According to Caldwell, et al (1998) [14], adolescence is a "...postpubertal population younger than 20 years who have a distinct life style." They often want to discuss topics, such as physical fitness, stress, nutrition, STDs, HIV/AIDS, alcohol, good eating behaviors, and contraception with their councillors [15-17]. They also hesitate to request personal health information from their physicians [15]. Adolescents also struggle with lack of knowledge about reproductive health and healthy sexual relationships [18-20]. Adolescents in general are uncomfortable discussing private health issues, such as sexuality and contraception, and younger adolescents are embarrassed, afraid, or uncomfortable discussing certain health issues (e.g., menstruation, pregnancy) than were their older peers in grades 10 through 12 [15].

Considering the attributes of cheapness, availability, ease of use and confidentiality of online resources, adolescent information needs may better be served by the Internet, which allows them to explore sensitive topics online which they may not want to reveal to parents, physicians, school officials, or acquaintances [21]. Web resources such as web pages, bulletin boards, newsgroups, listservs, and chatrooms found on the Internet contain health information and provide access to information for a potentially large number of participants worldwide [22].

About one third (36.5 million) of Nigeria's total population of over 123 million are youth between the ages of 10 and 24. It is estimated that by 2025, the number of Nigerian youth will exceed 57 million. Lack of reproductive health information and services place these young people at risk of pregnancy, abortion, sexually transmitted infections (STI), and HIV/AIDS. In all communities, girls are more vulnerable to reproductive health problems than boys for both biological and social reasons, as well as for cultural and patriarchal factors, and they often have poor access to information about their reproductive health. Although youth in Nigeria generally face health challenges, the adolescent girls face serious challenges, ranging from early exposure to intercourse to pregnancy, with severe consequences of abortion and death, and poor development of self-efficacy [23]. Despite harsh political and economic situation in Nigeria, the country is rapidly evolving as an information society and an information technology hub in West Africa. Internet services are available in cybercafes, in-schools and in many homes, and could be serving the purpose of informing the girl child about her reproductive health. However, there is need to understand the extent to which this infrastructure serves this purpose.

## Methods

### Study area, sampling and procedures

This survey took place in Owerri, an ancient city in eastern Nigeria, which is also the capital city of Imo State. Owerri covers a wide geographical area, a factor that combines with the high cost of attempting to reach every adolescent girl and the need to be time-conscious to necessitate the adoption of a sample survey design, already described in [24]. The study is concerned with two groups of respondents, namely the in-school, referring to adolescent girls who are currently in-school, and, out-of-school adolescents – adolescents who dropped out-of-school without completing their secondary education. This paper is restricted to adolescent girls being an excerpt from a larger research report with same focus, conducted with funding support from MacArthur Foundation in 2005 [24].

To survey the in-school adolescent girls, the principals of ten of the 22 secondary schools spread in the nine local government areas that constitute Owerri senatorial zone were approached through community champions, and their cooperation in collecting data from the students was solicited. Following from this, attendance rosters from each of the school authorities were collected, and 120 adolescent girls whose ages were 19 and below were randomly selected to participate in the study. The respondents completed the questionnaire in their schools with the assistance of trained interviewers. For the out-of-school sample, the community champions were relied upon to recruit adolescent girls, and 134 gave their consent. The instrument was administered to the girls in their homes. Altogether, the sample size was 1334. A major limitation of the study is the incomparability of the proportions of in-school and out-of-school samples, a factor that might reduce the significance of the comparison. However, considering the difficulty of tracking the out-of-school youth, the data used here gives sufficient indication of the situation. To ensure that the study met ethical expectation, details of the objectives, rationale and methodology of the study were disclosed to the respondents after their verbal consent was obtained. Furthermore, anonymity of the respondents was ensured by requesting them not to write their names on the questionnaires.

### The instrument and measures

The instrument used in this study was a modified version of an earlier questionnaire used in a research carried out on U.S. adolescents on the same subject matter [25,26]. To ensure that the instrument suited the problem defined, staff of the Africa Regional Centre for Information Science of the University of Ibadan reviewed the instrument for validity. The reproductive health information content of the instrument was reviewed by a biostatistician who was also the mentor appointed by the funder of the larger project. The major difference between this instrument and that used in the US study is the adjustment of the instrument for cultural appropriateness. The question on ethnicity was removed entirely because it was assumed that the non-Igbo population in the sample would be negligible since the study took place in an Igbo enclave. With respect to socio-economic status, car, telephone, and refrigerators, which are household appliances that are significant indicators of both enlightenment and affluence, were included. Then a one-time-only 16-page anonymous questionnaire consisting of 65 closed-ended questions was administered on each of the respondents. A pre-test showed that each of the instruments took approximately 35 minutes to complete. Data on the characteristics of the respondents, as well as a brief assessment of the adolescent's general health behaviors, knowledge, and attitudes, socio-economic status with respect to whether the household had items such as a telephone etc, media ownership, use, and knowledge, including accessing online health information, and their perceptions of the Internet, were then collected. In general, multiple-choice questions were used, for which participants could easily indicate their responses. For example, a list of 20 topics was provided from which participants could check off those topics they had tried to get information on from the Internet. On a few occasions, an oral interview was used to clarify issues that were not clearly gauged from the questionnaire, an undertaking that has been fully described in Nwagwu [24]. The data was analysed descriptively first, and then Chi square was used for comparisons, placing our significance level at p < 0.05. Student's t test was used to compare the perception of the in-school and out-of-school girls regarding the quality of information resources they found on the Internet. Regression analysis was further used to gauge how some reproductive health issues for which the adolescents used Internet could be predicted by their demographic characteristics.

## Result

Out of the 1200 copies of questionnaire distributed to the in-school adolescents, 1129 were returned, with 1011 being usable. This high return rate, which represents 94% and 84% respectively, was achieved mainly because the school authorities were involved in mobilizing the participation of the students. When the number of out-of-school students was added, there was a total of 1145 respondents. The personal characteristics of the respondents are shown in Table 1. The Chi square values, which are significant for all the characteristics in the two groups, underpin some differences between the in-school and the out-of-school girls.

**Table 1:** Some characteristic of the respondents

**Characteristics**	**Total** N = 1145	**In-school** n = 1011	**Out-of-school** n = 134	**Chi Square statistic**
**Age (%)**				67.12**
13	14.12	14.44	12.12	
14	17.23	17.33	13.08	
15	16.23	16.11	17.94	
16	13.08	15.18	12.01	
17	12.19	14.45	13.13	
18	16.18	12.26	16.43	
19	12.17	11.23	15.33	
**Living with (%)**				121.12**
Parents	37.39	32.01	47.15	
School Boarding house	12.17	15.48	5.95	
Other Hostel	22.78	12.5	28.02	
Others	27.66	40.01	28.88	
**Education status of parents (%)**				109.09**
None	12.13	4.16	15.21	
Primary	14.33	15.14	17.32	
Secondary	30.12	21.18	35.12	
College of education/Polytechnic	15.43	12.33	19.00	
University	34.09	47.19	13.35	
**Occupation of parents (%)**				12.09**
Self employed	23.17	19.19	24.13	
Private sector	34.15	45.13	25.26	
Public sector	42.68	35.68	50.61	
**Household appliances (**%**Yes)**				
Radio	79.11	81.22	76.71	68.68**
Television	78.21	79.11	76.81	78.42**
Telephone	22.18	23.48	21.10	89.16**
Car	43.67	45.27	32.12	33.58**
Computer	19.90	23.41	18.11	55.22**
Refrigerator	45.09	47.01	46.01	21.43**

** = significant at 0.05

Table 2 presents information concerning working radios, hours spent a week listening to radio, working televisions in the household and hours spent a week watching television as well as Internet use, Internet access at home and email accounts ownership for all the categories of respondents. A Chi Square analysis shows that at 5% level of significance, the two groups are also different in these use aspects of the selected media. These differences would therefore require that each of the groups be discussed differently.

**Table 2:** Aspects of use of selected media

	**Total** N = 1145	**In-school** n = 1011	**Out-of-school** n = 134	**Chi Square statistic**
Working radios in household (%)				
0	17.1	11.7	18.8	12.45**
1	65.9	66.0	76.6	
2 or more	17.0	22.3	4.6	
Hours a week listening to radio, Mean (SD)	21.13(12.56)	6(4.87)	23.34(18.18)	6.22**

Working televisions in household (%)				
0	14.8	9.9	15.5	67.22**
1	69.5	73.9	75.3	
2 or more	15.7	16.2	9.2	
Hours a week watching television, Mean (SD)	19.45(13.90)	22.13(12.19)	11.98(22.89)	12.14**

Ever used the internet (%yes)	73.1	74.9	68.6	12.17**
Home access to the Internet (%yes)	41.9	43.9	5.6	22.19**
E-mail account (%yes)	58.0	65.2	57.4	1.99**

### The in-school adolescents

Majority of the in-school respondents have one radio and one television respectively in their household, and the average number of hours spent watching television is more than that spent listening to radios. Also, the number of girls that reported ever having used the Internet was more than those who reported having email accounts, and further much more than those who have access at home. The survey also asked questions regarding frequency of use of the Internet, and found that of those who have ever used the Internet, 15.6% had used the Internet zero days in the last one month, 25.6% had used the Internet 1–5 days in the last one month and 58.8% had used the Internet one to three days in the last one week. There is no significant relationship between frequency of use of the Internet and occupation of parents and household appliances. But there is a significant relationship with age, living type and educational status of parents. A surprising result is that those with home access reported that they used the Internet slightly less frequently, 49.59% having used the Internet two to three days in the last one week, than those without home access, with 50.41% using the facility during the same period. More surprising is the observation that those with home access reported having used the Internet more at the cybercafes (64.25%) than they have used their home facility (35.75%). Further investigation of the media through which those who have Internet facility at home established access, found that 12.24% used phone line, 34.23% used a cable modem while 43.53% accessed the Internet through DSL facility.

Majority of the in-school adolescents (72%) accessed the Internet services through the Internet cafes, 43% used the school facility, 15% home facility, while 13% and 9% respectively reported that they used Internet facilities in friends' homes and other family members. The reason for this pattern of access was investigated further by an oral interview, and it was discovered that the adolescents preferred locations where they would enjoy unsupervised access [24]

The issue of reproductive health information sources used by the adolescents is now addressed (Table 3). Among the in-school adolescents, parents, teachers, public health campaigns, books, health provider/clinics, and health classes in descending order, each constituted more than 50% of the sources through which the adolescents sourced health information. Those in-school adolescents who are living with their parents (22.23%) are almost as likely as those living in-school boarding houses (23.13%) and other hostel (24.23%) to use the Internet for reproductive health information purposes while those in other type of residential category have the most likelihood (31.41%). There is also a significant difference between the use of Internet for reproductive health information purposes with type of access. The adolescents who have home access (33.12%) are less likely than those without home access, just as those who have ever used the Internet (79.19%) are by far most likely than those who have never used the Internet to exploit the use of the facility for the purpose of searching for reproductive health information. All those who have ever used the Internet for reproductive health information purpose also reported having own email accounts.

**Table 3:** Reproductive health information sources used by the adolescents

**Sources**	**Total **(%)	**In-school **(%)	**Out-of-school **(%)	**Chi square statistics in-school vs. out-of-school**
Health provider/clinic	54.14	55.24	25.44	6.60**
Parents	56.11	66.22	26.13	22.10**
Books	45.56	55.35	46.16	7.12**
Public health campaigns	45.78	55.77	35.18	5.80**
Health class	50.51	51.98	35.11	5.12**
Teachers	44.15	56.15	24.15	8.78**
Television	39.18	19.12	41.08	45.10**
Internet	45.19	25.12	55.19*	10.08**
Friends	53.18	49.11	63.18*	45.89**
Magazines	39.18	19.13	41.08	44.09**
Clergy/religious leader	19.10	13.00	21.00	ns
Grandparents/relatives	19.19	11.99	29.10	ns
Siblings/cousin	19.89	21.81	19.08	34.12**
Boyfriend or girlfriend	43.91	41.77	52.51	Ns

This author joined Borzekowski *et al.*[25] to list 22 specific and reproductive health related topics, (20 topics in all), and asked the youths to select the ones on which they have ever sought for information. The topics listed are alcohol use, cancer, contraception, dating, violence or rape, diet/nutrition, drug use, fitness or exercise, heart disease, illness support groups, and medicines/pharmaceuticals. Others are mental health, parenting, physical abuse, pregnancy, puberty/development, sexual abuse, sexual activities, sexually transmitted diseases, and HIV/AIDS. A crucial issue that has high reproductive health consequence among the youth namely pornography was excluded from this list because such sensitive information might not be gauged from the youth by a questionnaire.

Table 4 shows the result on the top eight topics, indicating that that HIV/AIDS and sexually transmitted diseases attracted the in-school girls to the Internet the most, followed by sexually transmitted diseases and pregnancy.

**Table 4:** Proportion of in-school and out-of-school adolescents accessing selected health topics

**Health topics**	**Total Internet users **(%)	**In-school Internet users **(%)	**Out-of-school Internet users **(%)	**Chi square statistic**
Sexually transmitted diseases	35.17	34.12	54.22	14.19**
HIV/AIDS	45.16	48.19	38.10	ns
Diet/nutrition	14.11	25.39	15.19	ns
Fitness or exercise	21.10	11.11	21.16	ns
Sexual activities	36.98	18.19	38.09	34.12**
Pregnancy	32.30	23.19	33.11	23.18**
Drug abuse	12.10	19.89	9.08	ns
Sexual abuse	11.10	29.29	9.20	ns

To the extent that the result so far has shown that Internet could be considered a source of information about adolescent reproductive health, it becomes very necessary to understand why the adolescents would prefer using the Internet instead of other sources.

**Figure 1:**
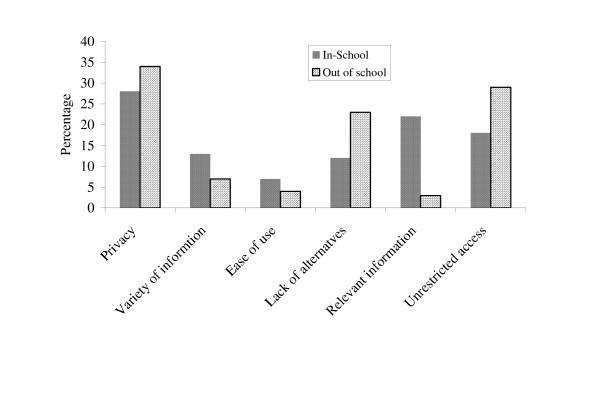
Why do you prefer the Internet?

Figure 1 shows that the in-school adolescents use the Internet for seeking reproductive health information because of its privacy, relevance of information freedom of access followed by lack of alternatives, variety of information and ease of use. Let us now turn to the out of school adolescents.

### The out-of-school adolescents

More than 68% of the out-of-school youth have ever used the Internet, with 5.6% having home access and about 57% reporting owning email accounts. Of all those who have ever used the Internet, 2.12% have sought for information from the Internet in the last one month, 45.12% have used the Internet 1–5 days in the last one month. Significant differences exist between frequency of use of the Internet by the out-of-school category and age, living status, educational status of parents as well as the occupation of parents. Over 23% of the out-of-school girl youth aged 15, 45.13% of those aged 18 and 37.71% of those aged 19-these age groups also constituted the largest proportion of respondents in the study (Table 1) – reported using the Internet for seeking information. Also, 34.32% of those living with their parents, 32.15% of those who reported living in boarding houses, 12.16% of those living in other types of hostel and 11.33% of those whose living statuses are other reported having used the Internet for information during the specified periods.

On the other hand, those out-of-school youth whose parents are public servants (33.10%) and have university education (33.19%) have used the Internet more than those whose parents are self employed (46.12%), employed by the private sector (20.78%) and those whose parents have secondary (28.19%) or college of education, (8.11%), primary (15.21%) and none (15.31%). There is no significant relationship between frequency of use of the Internet and availability of home appliances, although the probability appears higher for those who have computers at home (p = 0.051) than those who do not have (p = 0.031).

Those out-of-school adolescents who are living with their parents (12.56%) are less likely than those who reported living in boarding houses (27.13%), other hostel respondents (28.91%) and those who did not specify their accommodation types (31.41%) to use the Internet for reproductive health information. Unlike the in-school adolescents, there is a significant difference between the use of Internet for information purposes with type of access, with the adolescents who have home access (23.12%) being less likely than those without home access. A further significant difference exists with ever using the Internet, with an indication that, those who reported having ever used the Internet (87.13%) being by more users of the Internet for reproductive health than those who reported having never used the Internet. Similarly, owning an email account is significantly related to using the Internet for reproductive health information seeking purpose, with those reporting that they own email account also reporting using the Internet for reproductive health information seeking.

Cybercafes are the most patronized Internet service location for most of the out-of-school adolescents as 95.23% of them reported that they have used such location; the rest have used home facility (2.19%), school facility (0.2%), and 44.15% and 15.23% have used Internet in friends and other family members homes respectively. There is no significant association between age, availability of home appliances and the Internet location of choice. But youth whose parents are public servants (54.34%) differ from those whose parents are private sector employees (23.15%) or self-employed (12.51%) in their choice of Internet location. Furthermore, children of parents with tertiary education (15.18%) are less likely to choosy of Internet location, than those whose parents have secondary school education (24.15%), primary (34.12%) or none (22.55%). An examination of this response pattern showed that privacy, ease of access, unrestricted access and lack of alternative sources determined the choice of location.

What are the common reproductive health information sources used by the out-of-school adolescents? The out-of-school adolescents reported relying on friends (63.18%) the most for reproductive health information followed by the Internet and boyfriend/girlfriend each of which was reportedly used by over 50% of the respondents. Books, television and magazines were used by over 40%, while public health campaigns and health classes constituted sources of information to more than 30% of the respondents. Grandparents, parents, health provider/clinic, teachers and clergy/religious leaders were only reportedly used by more than 20% of the girls while sibling/cousin were the least consulted sources (Table 3). No significant relationship was found between the variables and personal variables of age, and, availability of household appliances. But significant differences were found with reproductive health information-related information sources except, surprisingly, clergy/religious leader, grandparents/relatives and sibling/cousin.

Unlike their in-school counterpart, there is a significant relationship between age, parents' occupation and living status. Adolescents at ages 18 and 19 are most likely (67.14%) to use the Internet for reproductive health information purposes than those who are less than 18 years. Those whose parents have tertiary education (45.18%) and who are living in other hostel accommodation (44.23%) are more likely than those whose parents have secondary education (21.11%) or primary education (33.79%) and who live in undisclosed hostel types (12.10%) or school hostel (2.10%) to use the Internet for health information purposes.

Type of access also differed with the use of Internet by the out-of-school youths. Access to the Internet at the cybercafés (56.12%) more than home based services (31.11%) is more geared towards using the Internet for reproductive health information searching. All the respondents who have ever used the Internet (34.12%) and who have email accounts (29.09%) also reported having used the Internet for seeking reproductive health information.

Regarding why the adolescents would prefer using the Internet instead of other sources, the response to this question is presented in Figure 1, and it shows that the out-of-school adolescents want an information source that they could consider private in addition to the fact that they considered Internet as having unrestricted access followed by lack of alternatives. Variety of information, ease of use and the relevance of information are the least. Privacy was found to be significantly associated with age, living status and adolescents older than 16 years (59.19%) compared with the younger ones (40.81%) considered the privacy factor an influence to their choice of Internet as a health information source. Adolescents who are living with their parents (36.15%), in-school boarding houses (24.10%), other hostel types (20.34%) and other types of living statuses (19.31%) also would prefer the Internet because of its privacy attribute. The children of the public servant parents (17.19%) less than those of self-employed parents (47.62%) and those of private sector employee parents (45.19%) shared this same view. None of the other characteristics of the respondents was significantly related to the privacy attribute of the Internet.

The health topics that most attracted the out-of-school youth to the Internet were the need to get information about sexually transmitted diseases, HIV/AIDS and sexual activities (Table 4). Apart from other category of needs, the youths seem to be less bordered about pregnancy-related information as well as fitness and exercise, but not as much for drug and sexual abuse and diet/nutrition. Out-of-school of various parental occupational categories also showed significant differences in the health topics for which they used the Internet. For instance, adolescents younger than 16 expressed more interest on HIV/AIDS (56.15%) and sexually transmitted diseases (66.35%) information than their older sisters, 43.85% and 33.75% respectively, but no significant differences were established for the others. There is also a significant relationship between the use of the Internet for sexual activities information with those youths whose parents are public servants (34.12%), self-employed (29.20%) or private sector-employed (36.68%). Let us now compare the in-school youth with the out-of-school ones in respect of the key issues that are emerging in this study.

#### Regression analysis result

Using regression analysis, some reproductive health issues for which the adolescents use Internet for information were predicted using the respondents' demographic characteristics, and the result is shown in Table 5. The slopes (B) show the extent of change on the dependent variables (reproductive health issues) that could be accounted for by a unit change in the independent variables (demographic variables), while the Se signifies the standard error of the regression coefficient. Negative or positive slopes therefore indicated the direction of this change. On this account therefore, Tables 5 and 6 show that living status of the students made positive, although fractional and significant contribution on the use of the Internet to seek for information about gay/lesbian/bisexual and STD issues. On the other hand, living status made negative but significant contributions on use of the Internet for information seeking about physical changes, as well as on contraception, pregnancy and parenting. Age positively predicts physical changes, as well as pregnancy and parenting but negatively predicts contraception, STD, and Gay/lesbian or bisexual issues. School status predicts physical changes, contraception, STD, gay/lesbian/bisexual issues, pregnancy and parenting with negative values for the last two variables, but the prediction for parenting is not significant. Educational status of parents, household appliances and occupational status of parents consistently positively predicted all the reproductive health information use of the Internet although educational status of parents and pregnancy, educational status of parents and pregnancy are not significant.

**Table 5:** A regression analysis of interest in using Internet for health information

	**Living status**			**Age**			**School status**		
	
	B	Se	P	B	Se	P	B	Se	P
Physical changes	-0.01	0.13	0.01	0.21	0.32	0.001	1.11	0.02	0.001
Contraception	-0.34	0.11	0.03	-1.14	0.21	0.001	1.31	0.01	0.001
STD	0.08	0.21	0.04	-1.8	0.19	0.001	0.18	0.10	0.001
Gay, lesbian or bisexual issues	0.41	0.18	0.02	-1.11	0.11	0.001	3.40	0.01	0.001
Pregnancy	-2.91	0.08	0.01	1.01	0.02	0.001	-2.31	0.01	0.001
Parenting	-1.34	2.11	0.01	3.11	0.90	0.001	-2.14	0.19	Ns

**Table 6:** A regression analysis of issues of interest in using Internet for health information

	**Educational status of parents**			**Household appliance**			**Occupational status**		
	
	B	SE	P	B	SE	P	B	SE	P
Physical changes	0.21	0.23	0.01	1.42	0.13	0.01	0.32	0.22	0.001
Contraception	0.21	0.21	0.01	0.42	0.13	ns	0.32	0.23	0.001
STD	0.28	0.21	0.01	1.29	0.19	0.01	2.21	0.39	ns
Gay, lesbian or bisexual issues	0.21	0.28	0.01	0.32	0.18	0.01	1.31	0.34	0.001
Pregnancy	2.82	0.28	ns	2.82	0.08	ns	1.72	0.23	0.001
Parenting	0.31	0.21	0.01	0.38	0.11	0.01	0.38	0.35	0.001

#### Comparing the two groups

Based on the data presented so far, a comparison can be made between in-school and out-of-school adolescent girls regarding using the internet to seek for reproductive health information. A crucial difference between the two groups relates to the sources of information they used. Table 3 shows that the top three sources used by the in-school girls are parents, teachers and public health campaigns whereas the out-of-school resorted to friends, Internet and boyfriends/girlfriend. The in-school resorted to grandparents (11.99%) the least in comparison with the out-of-school (29.1%), although this difference is reportedly not significant. While the Internet ranked ninth for the in-school, it ranked third for the out-of-school.

Regarding health topics for which the adolescents sought information, Table 4 shows that the in-school sought information about HIV/AIDS, sexually transmitted diseases and sexual abuse the most compared with the out-of-school whose information needs focused on sexually transmitted diseases, HIV/AIDS and sexual activities but the difference between HIV/AIDS and sexual abuse is not significant. The in-school sought information the least about fitness and exercise unlike the out-of-school who were least concerned about drug abuse.

Using a multivariate modeling, Table 5 shows that school status continuously predicted all the listed variables except parenting. The in-school also reported being more capable in finding information in the Internet than the out-of-school, despite reporting using the facility for reproductive health information the least. Also previous Internet use was associated with more confidence in using the Internet for reproductive health information more for the in-school than for the out-of-school. Table 7 shows that the two groups varied with respect to their perception about the quality of health information found in the Internet, with the in-school reporting the information as trustworthy and relevant more than the out-of-school who rather found the information as accurate and evident. Despite the fact that the out-of-school are wont to use the Internet for reproductive health information more than the in-school, Table 7 shows that the in-school assessed information in the Internet to be of higher quality than the out-of-school did.

**Table 7:** Perception about health information on the Internet

	**Total** (N = 1145)		**In-school** (n = 1011)		**Out-of-school** (n = 134)		**Students t-statistic**
	**M**	**SD**	**M**	**SD**	**M**	**SD**	
Useful	6.8	2.9	12.1	5.9	2.2	3.2	1.1
Easy to read	6.7	2.5	11.1	5.5	1.3	3.2	ns
Trustworthy	4.9	2.5	14.1	5.5	4.1	4.1	ns
Relevant	3.3	2.4	13.0	5.4	3.9	4.0	5.5
Accurate	5.9	2.3	9.1	5.3	7.1	2.0	ns
Visually appealing	5.4	2.9	5.4	4.9	4.3	4.8	ns
Source is evident	6.9	2.8	5.9	8.8	6.1	5.7	4.5

## Discussion

This study reveals that although the girl youth reported using the Internet in their quest to be informed about their reproductive health, the Internet serves the out-of-school more than they do the in-school. This fact holds irrespective of their living status, occupational and educational status of parents, age of the youth and availability of household appliances in their homes. In 2002, 90% of the youths in the US reported having gone online once [26], 63% have done this in Ghana in 2004 [25], whereas 74% of youth in this study have ever used the Internet. Our result shows, as in Ghana, that despite the fact that out-of-school adolescents had much less access to the Internet, the Internet was a relatively more important source of information for reproductive health information for them as shown in Tables 2 and 3, although they did not consider the information highly as did their in-school counterpart. This fact is clarified by a student t test in Table 7 which compares the mean perceptions of the two categories. The Table shows that the mean perceptions of the in-school adolescents were in all the cases higher than those of out-of-school while the reverse was the case regarding the standard deviations although the differences are only significant for 'useful', 'relevant' and 'source is evident'.

**Figure 2:**
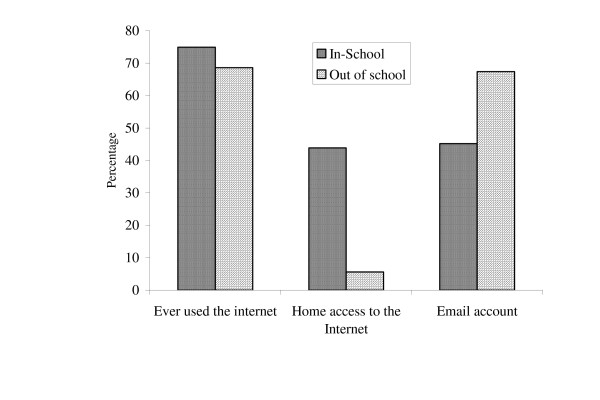
Aspects of media access.

The differential in the pattern of information sources reportedly available to the in-school and out-of-school youth is very interesting. Despite the relative advantageous home access which the in-school youth reportedly have over their out-of-school counterpart (Figure 2), the out-of-school youth use more of the Internet than the in-school, although they do not consider it a very qualitative source of information for their Parents and teachers, which are expected to be good sources of reproductive health information, seem to be mainly available to their in-school wards whereas friends and the Internet are the sources the out-of-school youth use most. This may explain why the out-of-school youth rely on private information sources for reproductive health information. Although this observation has some negative aspects and consequences on the out-of-school youth, particularly in respect of youth tendency towards sex experimentation, there could be a positive aspect to it. Cybercafes can become a veritable source of information about sex, STD and HIV/AIDS matters. If government and other agencies take relevant steps regarding restriction of access to pornographic and other devious sites, the cybercafés could be great sources of health information to adolescents. Very significant also is the relative non-relevance of the churches, elders and cousins/relations in acting as information providers for the youth.

The relative high tendency for school Internet access will not be unrelated to educational use of school Internet facilities in the school, which may be compulsory. On the other hand, the low report of use of Internet at home might be associated with technical, and, or use related oversight that might be mounted by parents and senior ones regarding what, when and why the adolescents would want to use the Internet as well as the fact that a high proportion of the youth were in-school. There is the question of power supply which is very erratic, and which might discourage home usage more than the usage of client-based commercial Internet cafes where alternative power systems are often available. Parents, teachers, books, and health provider/clinics are reportedly available as sources of reproductive health information to the in-school youth as could be inferred from Table 3. The out-of-school youth are not as lucky as they tend to rely mainly on their friends, the Internet and their boyfriends for reproductive health information. The non-availability of parents as sources of information to the out-of-school girls might be because of the low level their parental educational status. Moreover, some proportion of the out-of-school reported not living with their parents, a situation that might justify the parent-child communication gap.

There is also a low role for television and magazines for the in-school youth, sources which are very much used by the out-of-school. For the two groups, the role of the church/clergy as well as those of siblings and cousins is also considerably very low. More and more, the Internet services offered in the cybercafes are becoming very popular, inexpensive, readily available, and most significantly providing hideout for youth to browse those categories of information that should normally require supervision. A scan of the major city of Owerri where this study was carried out showed that most of the cybercafes constructed cubicles around computer nodes; a feature, which ensures that privacy of access and use, are guaranteed for every browser. Many cybercafes are for-business outfits whose major objective is to make money.

It is a popular observation that parents do not tend to communicate about reproductive health with their children in most African communities, due to "illiteracy" and "cultural practices", and that adolescents might therefore "tend to rely on informal sources for information about their sexuality." This study shows however that in comparison with other sources, parents appear to be the most frequently cited source of information about reproductive health among adolescents, particularly for the in-school girls.

A crucial question that emerges in this study is whether the Internet is actually an appropriate way to reach adolescent girls in Owerri and probably in other urban cities in Nigeria? This question arises in view of the fact that the study suggests that most of the study participants do not primarily use the Internet for reproductive health information. The list of the study participants who have sought on-line information on various topics does not show that even 50% of respondents indicated seeking online information on any of these topics (Table 4).

As in all communities, the number of channels available might limit the utility of televisions and radios, just as telephone might not provide visual and other accoutrements necessary to enhance engagement and interactivity. But the Internet gives the girl a high degree of interactivity, offers an anonymous, non-punitive, confidential and easily accessible space to find sensitive information. Moreover, the commercial based services often guarantee security and privacy of information; are cheap, available and accessible, and the skill required to use the service is no more difficult to acquire.

Using the Internet for information seeking is also however fraught with many dangers and could be counterproductive for youth when unguided. First, adolescents are still at stages in life when they require guidance regarding the choices they make about their health. An unguided reliance on the Net denies parents and other care givers the opportunity to vet and control the information their daughters receive from the chaotic nature of information content of the Internet. Furthermore, an unguided reliance on the Net for health information sidelines parents and teachers and other care givers who sometimes possess skills that are more suitable, both in curriculum and in local content, for adolescent education.

However, Internet, especially when located in the home, could create a new role for care providers, parents and teachers in informing and counseling as well as guiding young people's information choices. But the low use of home Internet for information reported here might mean that parents and adults have not incorporated the Internet as a strategy for adolescent health education. Adequate enlightenment is required to break information and communication gaps that exist between parents and children regarding using the Internet for reproductive health issues.

Information professionals and other practitioners should accord the Internet a higher place as a health information resource, which however requires a guided access when youth people use it. As the technology continues to spread to the rural communities, it seems logical to suggest that pro-health Web sites addressing different health needs of the youth who are the major patrons of the cybercafés should be developed and enforced. This is because the youth have some good levels of access and interest but not necessarily confidence, and favourable perceptions regarding the Internet as a source of health information. A crucial issue in these regards would be an expression of consciousness by the relevant government agencies and non-government organisations regarding design and implementation of appropriate community portals that address issues.

Designers and content providers might also consider exploiting the current deep and fast penetrating and versatile Web 2.0 technologies which are more youth-friendly by incorporating reproductive health information in blogs, wikkis, podcasts, and other web 2 facilities, which are now ubiquitous in the Internet [27]. A crucial question regarding why the out-of-school youth express a relatively high preference for the Internet as a source of information about their health needs to be addressed. Out-of-school girls might want to be in active control of their behaviours, and would often want to choose what they consider as useful to them more than the in-school.

Although the adolescent girls actually reported awareness that other sources of information exist, the out-of-school use the Internet mainly because of its privacy. The initiative of the out-of-school adolescents to link gratification of their personal needs with the media is based on the assumption that the media user is able to make accurate decision regarding what site, select the information and then use it. This may explain why the out-of-school adolescents assume that they are sufficiently knowledgeable about what they needs. Herein then also lies the difficulty of deploying the Internet for the purpose of availing adolescent reproductive heath information. In addition to consisting aspects that individual youth can go explore on their own, there are other aspects of the Internet that require the intervention of experts and senior members of the family and society. Adolescents are at the early stages of their development, and may, from time to time, require human expert guidance regarding their reproductive health life. It becomes very necessary to know what the out-of-school adolescents who inquire about their reproductive health from the Internet do with the information they obtain. Does such information lead them to make further inquiries from health personnel, parents or senior ones and other sources? Or does it lead them to devise private means of helping themselves when they find themselves in trouble?

Finally, this study raises some methodological questions. For instance, the specific sources of information referred to as "the Internet" are not defined, thus taking for granted the possibility that not all the respondents might be skillful in surfing the web for reproductive health information. The very low percentages of girls in this study who perceived information gleaned on the Internet as high quality leads one to ask from which of the myriads of reproductive health websites from which the girls actually sought information. However, there exist, in addition, local disabling factors such as erratic power supply, low level of penetration, low level of Internet literacy as well as cultural factors which may obstruct the significance of the Internet for reproductive health information among the girls in the city. Another relates to the fact that using questionnaire to study reproductive health information use of the Internet might be tricky because the respondent could be telling the researcher what the researcher might want to hear. But getting around these problems is very difficult – one alternative would require creating logs in the servers of the cafes where the adolescents go surfing for information, and analyzing this later – a very unethical technique. If there is any opportunity for further study on this issue, adequate training of the respondents regarding use of the Internet, the objectives of the study as well as increased number of out-of-school might reduce the difficulties of generalising the result presented here [24].

## Conclusion

This study has revealed the extent to which in-school and out-of-school adolescent girl youth in Owerri, an ancient community and capital of Imo State in Nigeria are using the Internet to meet their information needs. The study has also shown the extent to which the Internet could serve as a reproductive health information resource to both in-school and out-of-school adolescents. There is a wide disparity in the characteristics of the two categories of respondents in the study, and this informed the separate examination of the variables. Although the two groups are incomparable in terms of magnitude, the study gives an indication that in-school and out-of-school adolescents might be facing different information challenges as shown in their different patterns of use of the global infrastructure. Further studies are required to understand how to integrate parental and other care givers' roles in adolescent's use of the Internet for health information seeking, and also to understand why home-based Internet access serves adolescents other than reproductive health information purposes.

## Competing interests

The author(s) declare that they have no competing interests.

## Pre-publication history

The pre-publication history for this paper can be accessed here:



## References

[B1] Alubo O (2000). Research paper No. 166. Takemi Program in International Health, Harvard School of Public Health, 665, Huntington Avenue, Boston, MA 02115.

[B2] Katchadourian H, Feldman SS, Elliott GR (1990). Sexuality. At the threshold: The developing adolescent.

[B3] Moore S, Rosenthal D (1993). Sexuality in Adolescence.

[B4] United Nations Development Programme (2000, April) (2000). Report of the meeting of the high-level panel of experts on information and communication technology. New York.

[B5] Edejer TT (2003). Disseminating health information in developing countries: the role of the Internet. BMJ.

[B6] Odutola AB (2003). Developing countries must invest in access to information for health improvements. J Med Internet Res.

[B7] Arunachalam S (1998). Assuring quality and relevance of Internet information in the real world. BMJ.

[B8] Borzekowski DLG, Rickert VI (2001). Adolescents, the Internet, and health:Issues of access and content. Journal of Applied Developmental Psychology.

[B9] Gould MS, Munfakh JLH, Lubell K, Kleinman M, Parker S (2002). Seeking help from the Internet during adolescence. Journal of the American Academy of Child and Adolescent Psychiatry.

[B10] Gray NJ, Klein JD, Noyce PR, Sesselberg TS, Cantrill JA (2005). Health information-seeking behaviour in adolescence: The place of the Internet. Social Science & Medicine.

[B11] Blumler JG, Katz E (1974). The uses of mass communications: Current perspectives on gratifications research.

[B12] Papacharissi Z, Rubin AM (2001). Predictors of Internet use. Journal of Broadcasting and Electronic Media.

[B13] (1994). International Conference on Population and development, Cairo Egypt. http://www.un.org/popin/icpd2.htm.

[B14] Caldwell J (1998). The Construction of Adolescence in a Changing World. Studies in Family Planning.

[B15] Ackard DM, Neumark-Sztainer D (2001). Health care information sources for adolescents: Age and gender differences on use, concerns, and needs. Journal of Adolescent Health.

[B16] Joffe A, Radius S, Gall M (1988). Health counseling for adolescents: What they want, what they get, and who gives it. Pediatrics.

[B17] Rideout V (2000). Generation Rx.com: How young people use the Internet for health information.

[B18] Andrew G, Patel V, Ramakrishna J (2003). Sex, studies, or strife? What to integrate in adolescent health services. Reprod Health Matters.

[B19] Banister E, Schreiber R (2001). Young women's health concerns: Revealing paradox. Health Care for Women International.

[B20] Borzekowski DL, Rickert VI (2001). Adolescent cybersurfing for health information: a new resource that crosses barriers. Arch Pediatr Adolesc Med.

[B21] McKenna KYA, Bargh JA (2000). Plan 9 from cyberspace: The implications of the Internet for personality and social psychology. Personality and Social Psychology Review.

[B22] White M, Dorman SM (2001). Receiving social support online: Implications for health education. Health Education Research.

[B23] Lou Q, Zhao E, Gao I, Shah (2001). Can the Internet Be Used Effectively to Provide Sex Education to Young People in China?. Journal of Adolescent Health.

[B24] Nwagwu W (2005). Di Nwanna and the reproductive heath of the girl child in Imo State Final report (Number 0483064000GSS).

[B25] Borzekowski LG, Fobil JN, Asante KO (2006). Online Access by Adolescents in Accra: Ghanaian Teens' Use of the Internet for Health Information. Developmental Psychology.

[B26] Borzekowski DLG, Rickert VI, Calvert SL, Jordan AB, Cocking RR (2002). Adolescents, the Internet, and health:Issues of access and content. Children in the digital age.

[B27] Boulos MN, Maramba I, Wheeler S (2006). Wikis, blogs and podcasts. a new generation of Web-based tools for virtual collaborative clinical practice and education. BMC Medical Education.

